# Electronic health record and primary care physician self-reported quality of care: a multilevel study in China

**DOI:** 10.1080/16549716.2023.2301195

**Published:** 2024-01-11

**Authors:** Wenhua Wang, Mengyao Li, Katya Loban, Jinnan Zhang, Xiaolin Wei, Rebecca Mitchel

**Affiliations:** a School of Public Policy and Administration, Xi’an Jiaotong University, Xi’an, PR China; b Research Institute of the McGill University Health Centre, McGill University, Montreal, Canada; c Dalla Lana School of Public Health, University of Toronto, Toronto, Canada; d Health and Wellbeing Research Unit (HoWRU), Macquarie Business School, Macquarie University, Sydney, Australia; e Newcastle Business School, University of Newcastle, Newcastle, Australia

**Keywords:** Electronic health record, quality of care, health information technology, primary care, clinical quality

## Abstract

**Background:**

Health information technology is one of the building blocks of a high-performing health system. However, the evidence regarding the influence of an electronic health record (EHR) on the quality of care remains mixed, especially in low- and middle-income countries.

**Objective:**

This study examines the association between greater EHR functionality and primary care physician self-reported quality of care.

**Methods:**

A total of 224 primary care physicians from 38 community health centres (CHCs) in four large Chinese cities participated in a cross-sectional survey to assess CHC care quality. Each CHC director scored their CHC’s EHR functionality on the availability of ten typical features covering health information, data, results management, patient access, and clinical decision support. Data analysis utilised hierarchical linear modelling.

**Results:**

The availability of five EHR features was positively associated with physician self-reported clinical quality: share records online with providers outside the practice (β = 0.276, *p* = 0.04), access records online by the patient (β = 0.325, *p* = 0.04), alert provider of potential prescription problems (β = 0.353, *p* = 0.04), send the patient reminders for care (β = 0.419, *p* = 0.003), and list patients by diagnosis or health risk (β = 0.282, *p* = 0.04). However, no association was found between specific features availability or total features score and physician self-reported preventive quality.

**Conclusions:**

This study provides evidence that the availability of EHR systems, and specific features of these systems, was positively associated with physician self-reported quality of care in these 38 CHCs. Future longitudinal studies focused on standardised quality metrics, and designed to control known confounding variables, will further inform quality improvement efforts in primary care.

## Introduction

Health information technologies, particularly electronic health record (EHR), have the potential to improve the delivery of health services and are considered to be among the critical building blocks of a high-performing health system [1–3]. There is evidence to show the positive impact of EHR on the workload of healthcare providers [4,5], on health outcomes [2,6], and healthcare processes such as patient-provider communication [7]. During the past decades, the adoption of EHR has been promoted as an important approach to improving the quality of healthcare by industry, physicians, and policy-makers, especially in primary care settings [1,8,9].

In high-income countries, the relationship between EHR and quality of care in ambulatory settings has been extensively studied since the 2000s. The first wave of studies focused on examining a basic association between any EHR implementation and quality of care, mostly using cross-sectional survey data. The findings from these studies were ambiguous, with several studies failing to demonstrate any relationship between the use of EHR and specific quality of care metrics (e.g. receiving appropriate therapy for chronic conditions or appropriate screening tests) [10–13], while other studies found modest effects on technical quality of care of chronic conditions [14,15]. A second wave of studies started to examine the association of specific components of EHR with different aspects of care. For example, the availability and use of specific EHR features by primary care physicians (PCPs) were associated with better performance on certain quality measures [11,16]. Electronic laboratory result viewing and the presence of a computer decision support and electronic problem lists were associated with higher ambulatory care quality [17,18]. More recent studies using longitudinal analyses or randomised controlled trials found that ambulatory clinics with EHR had better composite measures of clinical quality than other clinics [8]. Interventions using EHR in primary care led to minor improvements in blood pressure and lipid control, significant effectiveness in increasing physical activity, and meaningful improvements in preventive care [19,20]. This evidence supports the potential of EHR to improve the quality of care in primary care.

EHRs are increasingly implemented within low- and middle-income country (LMIC) settings to address several challenges in primary care especially related to care quality variability [9,21]. The limited and inconsistent use of EHR systems contributes to challenges related to both the safety and quality of health care [22]. Barriers to EHR adoption include poor infrastructure, lack of management capacity, organisational standards, interoperability, and user inexperience [23]. There is also compelling evidence that information systems need to be carefully specified for a given context [24], especially given the variable change management capacities of local health care organisations [22].

Since 2009, China has made a strong commitment to build the National Electronic Health Information System in order to support service delivery reform. Several studies of existing EHRs have recently been conducted in the hospital sector [25–27]. A 2017 cross-sectional national survey of EHR among 462 community health centres (CHCs) found that less than 59% of CHCs had adopted EHR, even with limited functions, although almost 90% of the health professionals in primary care believed that it was necessary to implement EHR in community health services, especially the features of clinical decision support (CDS) and sharing of information across organisations [28]. Further, in 2016, it was reported that there was a lack of assessment of the overall impact of EHR in primary care [29]. In order to guide further EHR systems building in primary care in China, this study examined the association of EHR functionality in general, and 10 specific EHR features in particular, on quality of care in 38 CHCs in four large cities in urban China.

## Methods

### Study setting

Four large cities in China were selected for this study: Shanghai, Shenzhen, Tianjin, and Jinan. These cities have relatively well-developed primary care systems with a high level of infrastructure development and health professionals qualified in primary care. Recent findings suggest that health professionals in primary care from less developed western region in China were less likely to recognise the value of an EHR compared with those in the more developed eastern and central regions in China [28], which suggests that large cities are more likely to promote the meaningful adoption of an EHR. The results from this study could inform further studies with designs for more generalisable sampling frames, or designs with more statistical power to examine the effect of specific EHR features in primary care settings in China.

### Sampling and data collection

The field research was conducted between November 2021 and May 2022. We used a convenience sampling approach to select a total of 38 CHCs: 10 in Shanghai, 14 in Shenzhen, 8 in Tianjin, and 6 in Jinan. The sample size was determined based on the requirements of multilevel regression models and the feasibility of data collection during the pandemic. The selection of CHCs in each city was suggested by the local health bureau staff based on the practice size and service performance levels of CHCs in their respective catchment areas.

In each CHC, all PCPs on duty the day the researchers arrived were invited to complete a physician survey, which included information on their socio-demographic characteristics, professional attitudes such as organisational commitment, well-being conditions such as burnout, and self-reported quality of care. Each CHC director completed a primary care organisational survey, including information on organisational characteristics, such as human resources, ownership, learning orientation, organisational resilience, leadership styles, and EHR functionality.

After the research assistants explained the purpose of this study and the content of this survey, self-administered paper questionnaires were handed out to the participants. After the participants completed the questionnaire, they returned it to the research assistants directly in order to reduce the risk of external influence on responses. The research assistants would check if there was any missing information to ensure the completeness of each questionnaire. All 224 staff physicians present at the 38 CHCs completed the physician survey, and the 38 CHC directors completed the CHC organisational survey. The ethical approval for this research was obtained from the Ethics Committee of Xi’an Jiaotong University.

### Measures

#### Quality of care

Because the EHR systems and primary care quality standards in China are still rapidly developing, there are many barriers to collecting comparable, objective, quality of care information directly from the disparate EHR systems. Therefore, two physician survey-based indicators were used to measure the quality of care that they provided in each CHC. Quality of clinical care was measured using one item ‘Do you often use “evidence-based” treatment guidelines released by national or medical associations to treat patients with chronic disease’ with a 5-point Likert response scale from 1 representing never to 5 representing always [30]. Quality of preventive care related to smoking, alcohol, diet, and exercise, and was measured through four items: health risk assessments, referral to community programmes, individual counselling, and group counselling. Responses in each item ranged from 1 (never) to 5 (always). The average score of four items was the final score of preventive care quality [31]. The higher the score, the better the reported care quality.

#### EHR functions

The 38 CHC directors completed a companion organisational survey, including information on EHR functions. The EHR features used to measure EHR functionality in this study were translated and adapted from the health information technology section of the Commonwealth Fund International Health Policy Survey of Primary Care Physicians [30]. To ensure the applicability and feasibility in the Chinese context, we conducted consultations with senior primary care researchers, local primary care managers, and physicians. Finally, 10 features representing four core EHR functions essential to a functioning EHR (health information and data, results management, electronic communication and connectivity, decision support) were used [13]. These features are reported in Table 2. Each EHR feature was categorised as available or not available by CHC. The mean of the ten pre-selected EHR features available in a given CHC is the total EHR feature score for that CHC [30].

Control variables were included in the two surveys. Self-provided physician characteristics were age, gender, education, and tenure. Organisational characteristics provided by the directors included management type (government-managed CHC or public hospital-managed CHC), government accreditation (accredited by national or provincial authorities or not), medical equipment number (the number of medical equipment (with the value over $1,600 USD)), CHC staff count (number of staff in CHC), and per cent of CHC staff who are health professionals (physicians, nurses, etc.). These control variables were selected based on previous studies while considering the primary care context in China [10–18].

### Statistical analysis

First, descriptive statistical analysis was conducted to describe the characteristics of PCPs and CHCs. Next, we calculated the mean score of both physician-reported clinical quality and preventive quality for the CHCs with available EHR features and CHCs without EHR features, for each of the ten specific EHR features. An independent sample t-test was used to compare the mean quality of care in CHCs with vs. without each specific EHR feature to determine if there was a significant mean quality difference associated with feature availability. Since the PCPs were nested within the CHCs, two-level hierarchical linear models (HLM) (physician at level 1; nested within the CHC at level 2) were built to examine the association of care quality with the availability of EHR features while taking into account the impact of control variables. Our sample size was sufficient for multilevel modelling, with a mean level 1 sample size of nearly six PCPs and a level 2 sample size of 38 CHCs. Reliability diagnosis of HLM models by calculating the Intraclass Correlation Coefficient (ICC) showed that the value of ICC for clinical quality and preventive quality was 0.086 and 0.145, respectively, indicating that 8.6% and 14.5% of the total variation in two types of quality of care occurred between CHCs and HLM modelling should be used. For each of the ten EHR features, two separate HLM models were built to examine its association with clinical quality and preventive quality while controlling for control variables, respectively. We also built two separate models to examine the association of total EHR feature score with clinical quality and preventive quality, respectively. Multicollinearity analysis using variance inflation factor also suggested no existence of multicollinearity in each model. All statistical analyses were performed using STATA 15.1.

## Results

### Physician and CHC characteristics

The responses of the 244 physician surveys (level 1) and 38 CHC surveys (level 2) are shown in Table 1. Of these physicians, 51.8% were under 35 years old, and less than 1% were over 54 years old. The majority of physicians (59.8%) were female, 16.1% had a master’s degree education or above, and 75.9% occupied officially tenured posts.

**Table 1.** Characteristics of physician respondents and CHCs.

Characteristics	n	%
Physicia* characteristics (*N* = 224)		
Age		
<35	116	51.8
35–44	75	33.5
45–54	31	13.8
>54	2	0.9
Gender		
Male	90	40.2
Education level		
Master or above	36	16.1
Tenured position		
Yes	170	75.9
**CHC characteristics (*N* = 38)**		
Management type		
Government	23	60.5
Public hospital	15	39.5
Government accreditation		
Yes	22	57.9
Medical equipment number		
<5	8	21.1
5–10	12	31.6
11–30	5	13.2
>30	13	34.2
Number of CHC staff		
≤35	13	34.2
36–55	7	18.4
56–100	7	18.4
>100	11	29.0

Note: *Physician refers to the 224 primary care physicians in the 38 CHCs. CHC: community health centre.

Of the 38 CHCs, 60.5% were managed by the government, and others were public hospital-managed. Over half were accredited by national or provincial authorities. Most CHCs had less than 35 staff. The percentage of CHC staff who were health professionals was 86.6%. In contrast to the national characteristics of CHCs and PCPs within CHCs in China in 2021, the sampled physicians were younger, with a higher education level and a higher percentage of females. However, the percentage of CHC staff who were health professionals closely paralleled the national average [32].

### Crude mean quality of care by specific EHR feature availability

Table 2 reports a) the availability of each of the ten EHR features among the 38 CHCs, with b) the associated means of the Likert scaled PCP self-reported quality of care, and c) the results of a t-test to detect a difference in the care quality score means between CHCs with and without each feature. More than half of the CHCs in our sample reported the availability of the following five EHR features: 2. Access records online by provider, 6. Alert provider to provide patients with test results, 7. Send the patient reminders for care, 8. List patients by diagnosis or health risk, and 9. List patients due for tests or preventive care. The availability of the other five features was reported by less than 50% of CHCs.

**Table 2.** Association of EHR feature availability with care quality as shown by t-test of difference in crude care quality means between CHCs with vs. without EHR features.

EHR features	Availability	Clinical quality	P value	Preventive quality	P value
1. Share records online with providers outside the practice	Yes (*n* = 17)	4.28	0.002^†^	3.71	0.041*
	No (*n* = 21)	3.88		3.47	
2. Access records online by provider	Yes (*n* = 24)	4.08	0.950	3.61	0.745
	No (*n* = 14)	4.08		3.57	
3. Access records online by the patient	Yes (*n* = 14)	4.21	0.006^†^	3.59	0.867
	No (*n* = 24)	3.86		3.57	
4. Access test results online by provider	Yes (*n* = 14)	4.11	0.581	3.61	0.648
	No (*n* = 24)	4.04		3.55	
5. Alert provider of potential prescription problems	Yes (*n* = 15)	4.18	0.043*	3.62	0.541
	No (*n* = 23)	3.92		3.54	
6. Alert provider to provide patients with test results	Yes (*n* = 20)	4.18	0.144	3.59	0.992
	No (*n* = 18)	3.99		3.59	
7. Send the patient reminders for care	Yes (*n* = 21)	4.30	0.006^†^	3.68	0.208
	No (*n* = 17)	3.94		3.53	
8. List patients by diagnosis or health risk	Yes (*n* = 22)	4.30	0.010*	3.68	0.221
	No (*n* = 16)	3.96		3.53	
9. List patients due for tests or preventive care	Yes (*n* = 21)	4.24	0.055	3.70	0.147
	No (*n* = 17)	3.99		3.52	
10. List of all medications taken by patients	Yes (*n* = 16)	4.10	0.775	3.63	0.424
	No (*n* = 22)	4.06		3.54	

Note: The Quality of Care Likert scale ranges from 1 representing worst to 5 representing best. The Quality of Care metric in this table is the mean value of the physician self-reported quality of care that they provide. EHR: electronic health record. CHC: community health centre.

*p < 0.05

^†^*p* < 0.01

In terms of clinical quality, PCPs working in CHCs with the following five EHR features reported a significantly higher mean care quality based on a t-test: 1. Share records online with providers outside the practice, 3. Access records online by the patient, 5. Alert provider of potential prescription problems, 7. Send the patient reminders for care, and 8. List patients by diagnosis or health risk. For preventive care quality, the differences in mean care quality between CHCs with a specific EHR feature vs. CHCs without the same EHR feature based on t-test were not statistically significant except for the following feature: sharing records online with provider outside the practice.

### Hierarchical linear regression models of EHR features and quality of care

Table 3 presents the strength of association, and statistical significance in the ‘fully controlled’ HLM models (which include all of the variables in Table 1) of the availability of each of the EHR features with both clinical and preventive quality. When modelled along, without other features in the model, a statistically significant, positive association with clinical quality is found for five of the ten EHR features: 1. Share records online with providers outside the practice (*β* = 0.276, *p* = 0.040), 3. Access records online by the patient (*β* = 0.325, *p* = 0.043), 5. Alert provider of potential prescription problems (*β* = 0.353, *p* = 0.037), 7. Send the patient reminders for care (*β* = 0.419, *p* = 0.003), and 8. List patients by diagnosis or health risk (*β* = 0.282, *p* = 0.035). Physicians working in CHCs with any one of the five EHR features reported significantly higher clinical quality scores compared to physicians working in CHCs without these features. These are the same features whose availability was identified as a significant factor in clinical care quality in Table 2 by t-test. However, the association between any one of the ten EHR features and preventive care quality was not statistically significant.

**Table 3.** Strength of association between ten EHR features and quality of care.

EHR features	Clinical quality		Preventive quality	
	*β (SE)*	*P*	*β (SE)*	*P*
1. Share records online with providers outside the practice	0.276 (0.135)	0.040*	0.032 (0.136)	0.816
2. Access records online by provider	0.431 (0.280)	0.123	− 0.123 (0.273)	0.652
3. Access records online by the patient	0.325 (0.160)	0.043*	− 0.021 (0.161)	0.896
4. Access test results online by provider	− 0.092 (0.144)	0.522	0.007 (0.142)	0.963
5. Alert provider of potential prescription problems	0.353 (0.169)	0.037*	0.191 (0.170)	0.262
6. Alert provider to provide patients with test results	0.104 (0.143)	0.469	− 0.053 (0.141)	0.708
7. Send the patient reminders for care	0.419 (0.142)	0.003^†^	0.028 (0.144)	0.844
8. List patients by diagnosis or health risk	0.282 (0.134)	0.035*	0.073 (0.135)	0.586
9. List patients due for tests or preventive care	0.190 (0.137)	0.165	0.033 (0.137)	0.807
10. List of all medications taken by patients	0.231 (0.150)	0.123	0.119 (0.149)	0.424

Note: EHR: electronic health record. Two models were built for each of the ten EHR features, one for clinical quality as dependent variable, and the other for preventive quality as dependent variable. For each model, control variables included age, gender, education, tenured position at physician level – level 1, management type, government accreditation, medical equipment number, CHC staff number, and per cent of health professionals at CHC level – level 2.

*p < 0.05

^†^*p* < 0.01

Table 4 shows that the total EHR feature score was strongly associated with clinical quality (*β* = 0.633, *p* = 0.010) but not associated with preventive care quality. In terms of covariates, age was positively associated with both clinical quality (*β* = 0.027, *p* = 0.001) and preventive quality (*β* = 0.018, *p* = 0.015), which suggested that senior physicians are more likely to rate care quality they provided higher. PCPs working in larger CHCs were likely to report lower care quality, while those in CHCs with more medical equipment were more likely to report higher care quality.

**Table 4.** Strength of association between total EHR feature score and quality of care.

Parameter	Clinical quality		Preventive quality	
	*β (SE)*	*P*	*β (SE)*	*P*
Intercept	2.763 (0.756)	<0.001^‡^	3.812 (0.754)	<0.001^‡^
**Physician**^¶^**characteristics – level 1**				
Age	0.027 (0.008)	0.001^‡^	0.018 (0.007)	0.015*
Gender (ref. = female)				
Male	0.154 (0.123)	0.210	−0.073 (0.112)	0.518
Education level (ref. = college or below)				
Master or above	−0.021 (0.178)	0.906	−0.019 (0.166)	0.911
Tenured position (ref. = no)				
Yes	−0.118 (0.173)	0.493	−0.031 (0.157)	0.843
**CHC characteristics – level 2**				
Management type (ref. = government)				
Public hospital	−0.144 (0.194)	0.458	0.073 (0.199)	0.712
Government accreditation (ref. = no)				
Yes	−0.168 (0.140)	0.229	−0.289 (0.145)	0.045*
Medical equipment number (ref. <5)				
5–10	0.183 (0.193)	0.345	0.024 (0.194)	0.900
11–30	0.346 (0.234)	0.139	−0.052 (0.238)	0.827
>30	0.521 (0.252)	0.039*	0.457 (0.258)	0.077
Number of CHC staff (ref. ≤35)				
36–55	−0.470 (0.194)	0.016*	−0.536 (0.199)	0.007^†^
56–100	−0.373 (0.217)	0.086	−0.583 (0.226)	0.010^†^
>100	−0.601 (0.210)	0.004^†^	−0.590 (0.220)	0.007^†^
Per cent of CHC staff who are health professionals	0.520 (0.634)	0.413	−0.590 (0.648)	0.363
**Total EHR feature score**	0.633 (0.246)	0.010*	0.103 (0.253)	0.683

Note: These quality scores were assessed on a scale of 1–5 (5 being best) by the 224 physician survey respondents who work at the respective CHCs. EHR: electronic health record.

*p < 0.05.

^†^*p* < 0.01.

^‡^*p* < 0.001.

^¶^Physician refers to the 224 primary care physicians serving the 38 CHCs.

## Discussion

Using data from 224 PCPs across 38 CHCs in four large cities in China, we found a statistically significant positive association between total EHR feature score and clinical quality as reported by PCPs. We also identified five specific EHR features which individually also had a statistically significant positive association with PCPs self-reported quality of care out of our ten single feature models; these were as follows: 1. Share records online with providers outside the practice, 3. Access records online by the patient, 5. Alert provider of potential prescription problems, 7. Send the patient reminders for care, and 8. List patients by diagnosis or health risk.

Our results are consistent with prior evidence suggesting that, to improve outpatient care quality, EHR developers, implementers, and certifiers could increase the adoption of the more highly featured EHR systems while improving and increasing the use of (or upgrading of) specific features, rather than aiming to deploy EHR regardless of functionality [11,33].

Sharing health information among different healthcare organisations has an important role in the healthcare system, especially in improving healthcare quality. By sharing health information among organisations, healthcare providers can immediately access patient information to make informed decisions regarding optimal treatment while preventing medical errors and adverse events and reducing readmissions and emergency room utilisation [34]. Recent studies have found that increased utilisation of community health information exchanges by physicians was significantly associated with reduced likelihood of visiting the emergency department and being re-hospitalised among their recently discharged patients [35], consistent with a previous study in China showing that a city-wide Health Information Exchange reduced redundant laboratory testing, procedures, and duplicate medications across the hospitals network [36].

This study appears to confirm a positive association of EHR sharing among multiple organisations with the quality of care as reported by PCPs across the 38 CHCs. China is developing a hospital–primary care organisation alliance model to boost service capacity, improving care quality and continuity of care in primary care settings with technical support from hospitals. Our findings are consistent with these reform efforts and support the reform efforts of building integrated health information systems to facilitate health information sharing between primary care and hospitals, especially in technical support and training, high quality of the information exchanged, and effective workflow improvements [37]. Further longitudinal cohort or randomised controlled trial (RCT) studies should be conducted to examine the effects of integrated health information systems implementations.

Providing patients with access to their EHR has been identified as an integral element in delivering patient-centred care. It may improve the quality of care by allowing patients to access their personal health information and involving them as key stakeholders in the self-management of their health [38]. Despite the arguments on the theorised benefits of providing patients with access to EHR, it is difficult to find evidence of their demonstrated impact, especially in LMICs and primary care settings [39]. Though evidence suggests that providing patients with access to EHR improves patient satisfaction and communication, no clear benefits were identified concerning care quality increase [38]. In the context of 38 sampling CHCs, our study showed a statistically significant positive association of providing patient access to EHR with higher quality of care as reported by PCPs.

As China is building the people-centred integrated care model, the use of information and communication technology, coupled with improving health literacy, strengthening self-management, and facilitating shared decision-making, is proposed as a core action area [40,41]. Further studies may be conducted to examine the effect and implementation of the feature of providing patient access to an EHR in a primary care setting. When introducing new public or professional use technology which supports complex health science communication and depends on multi-stakeholder engagement, a reasonably long learning curve should be distinguished from the absence of a desired effect.

CDS tools are widely recognised as very important approaches to facilitating quality improvement. CDS systems support clinicians in keeping up with the latest evidence in a smart way [42]. Electronic CDS tools are becoming increasingly available to assist PCPs with the diagnosis and management of a range of health conditions [43]. Many CDS tools exist for use in primary care and, more recently, are being embedded in patients’ EHR for their operation in high-income countries [43]. Computerised alerts and reminders within the EHR have been found to increase adherence to preventive care guidelines and improve management of chronic diseases. However, these studies were undertaken in high-income countries, which may limit generalisability in other areas [18] or which may simply indicate a learning curve delay and health consequences delay exist. This study suggested a positive association of EHR CDS features with quality of care reported by PCPs in a developing country setting. Previous studies suggest the value of understanding how system design, local context, implementation strategy, cost, and user experience influence the implementation of CDS tools [44], and the results of our study provide information that is of significant value for further health information system reform in China.

Accurate patient problem lists are valuable tools for improving the care quality, enabling CDS and facilitating research and quality measurement [45]. Improved problem list documentation has been associated with higher quality of care and greater adherence to evidence-based clinical guidelines [45]. Consistent with previous evidence in ambulatory care settings that found a positive association between problem lists and care quality in other countries [11,18], our study demonstrated a positive association in community primary care settings in a convenience sample of 38 CHCs in urban China. To promote meaningful use of problem lists, further research is needed to understand their implementation, the content of the lists, and policies that would enable adherence to these practices based on local context [46], which may also build knowledge of the associated learning curve and undesired effects of the potential burdens of EHR systems.

With regard to the five EHR features that were not associated with quality of care as reported by PCPs, we caution against the assumption that these are not important to optimal EHR systems. Our results can be explained by poor implementation including insufficient training, or simply insufficient time for the effects to manifest.

### Limitations

The following limitations should also be considered in interpreting our findings. First, this is a cross-sectional study, where the results can only confirm the association between EHR features and PCPs self-reported quality of care instead of demonstrating causation. Second, a cross-sectional design offers no protection against the effects of confounding variables not explicitly included in the models, e.g. ‘the extent of any non-use of available EHR features’ is a variable that was not available and not controlled in the model. Third, the 38 CHCs in four large cities were selected using a convenience sampling approach. Additionally, using only one feature per model excludes known significant feature variables, the omission of which may also confound results leading to unreliable betas and p-values for the specific feature in any such incomplete model. The results are applicable across these particular sampling sites and cannot necessarily be generalised to other primary care settings. Fourth, a subjective quality metric-physician self-reported quality of care was used to measure the primary care quality instead of objective quality metrics. This may introduce a potential bias risk as includes the PCPs’ own work; it is unknown whether the lack of preventive quality differences is related to the metric’s inaccuracy or lack of sensitivity, or to an actual non-effectiveness in this context. Fifth, the number of covariates in the HLM models may have ‘over-fitted’ the models, particularly given the small level 2 sample size of 38 CHCs. Subsequent work should be conducted to validate the results.

Despite the several limitations to this study that we carefully noted, we see a significant pattern in the 38 CHCs that we studied that is consistent with the broader literature that indicates EHR benefits in primary clinical care [8,9,19,20,47]. These limitations may be addressed by future studies, entailing probability sampling, a more data-intensive longitudinally designed study of EHR feature implementation and user training; a longer process of data collection and validation of functional elements of the EHR; analysis of case-level linkages between feature use and outcome(s) desired, particularly when patient data are spread across disparate EHR; documenting temporal relationships consistent with causality, and better control of potential bias and possible confounders.

### Practice implication

Digital technology including EHR has great potential to transform the health service delivery system by increasing efficiency and improving the quality of care. In this study, we found a positive association of EHR features with quality of care as reported by PCPs, across a convenience sample of 38 CHCs in four large Chinese cities. The experience and results from this study, as well as a careful review of the limitations which attend various study designs, could inform further, and more refined studies. These further studies would examine the effect, usability, and implementation of primary care EHR in China in order to inform further EHR system building and optimisation. While technologies and digital health have not yet reached maturity or wider acceptance as a means of improving primary care, stronger policy and financial support, as well as advocacy of key stakeholders, are needed to encourage the introduction of efficient EHR in order to support strong sustainable primary care [48].

## Conclusion

Across a convenience sample of 38 CHCs in four large Chinese cities, this study provides evidence that the availability of the certain EHR features is positively associated with physician self-reported quality of care in primary care. Further longitudinal (cohort or RCT) studies of the usability, implementation, and benefits of specific features of primary care EHR should be conducted in order to inform further health information system building in China.

## Paper context

Electronic health records have been promoted as an important approach to improving the healthcare quality, while the effect of its features in primary care is not clear. We found that features such as sharing health information among different healthcare organisations, as well as providing patients access to their electronic health records, are positively associated with primary care quality. Future electronic health records improvement should focus on information exchange between different institutes and enhancing patient engagement.
